# Predictors of Splenectomy Response in Immune Thrombocytopenia: A Multicentric Italian Study

**DOI:** 10.3390/jcm14010030

**Published:** 2024-12-25

**Authors:** Simone Zoletto, Marco Pizzi, Andrea De Crescenzo, Alberto Friziero, Fabio D’Amore, Giuseppe Carli, Nicola Vianelli, Giuseppe Auteri, Irene Bertozzi, Ilaria Nichele, Gianni Binotto, Angelo Paolo Dei Tos, Federico Scarmozzino, Emanuele S. G. D’Amore, Jessica Ceccato, Elena Sabattini, Francesco Cinetto, Francesco Piazza, Andrea Visentin, Renato Zambello, Livio Trentin, Fabrizio Vianello

**Affiliations:** 1 Hematology and Clinical Immunology Unit, Department of Medicine, University of Padua, 35122 Padua, Italy; simone.zoletto@gmail.com (S.Z.); andrea.decrescenzo1998@gmail.com (A.D.C.); fabio.damore@aopd.veneto.it (F.D.); gianni.binotto@aopd.veneto.it (G.B.); jessica.ceccato.1@phd.unipd.it (J.C.); francesco.piazza@unipd.it (F.P.); andrea.visentin@unipd.it (A.V.); r.zambello@unipd.it (R.Z.); livio.trentin@unipd.it (L.T.); 2 Surgical Pathology and Cytopathology Unit, Department of Medicine, University of Padua, 35122 Padua, Italy; marco.pizzi.1@unipd.it (M.P.); angelo.deitos@unipd.it (A.P.D.T.); federico.scarmozzino@aopd.veneto.it (F.S.); 3 Department of Surgery, Oncology and Gastroenterology, University of Padua, 35122 Padua, Italy; alberto.friziero@aopd.veneto.it; 4 Hematology Unit, San Bortolo Hospital, 36100 Vicenza, Italy; g.carli@aulss8.veneto.it; 5 Institute of Hematology, Sant’Orsola-Malpighi University Hospital, 40138 Bologna, Italy; nicola.vianelli@unibo.it (N.V.); giuseppe.auteri2@unibo.it (G.A.); 6 Department of Medicine-DIMED, University of Padua, 35122 Padua, Italy; irene.bertozzi@unipd.it; 7 Department of Cell Therapy and Hematology, San Bortolo Hospital, 36100 Vicenza, Italy; ilaria.nichele@aulss8.veneto.it; 8 Department of Pathological Anatomy, San Bortolo Hospital, 36100 Vicenza, Italy; emanuele.damore@gmail.com; 9 Haemolymphopathology Unit, Sant’Orsola-Malpighi University Hospital, 40138 Bologna, Italy; elena.sabattini@aosp.bo.it; 10 Rare Disease Referral Center, Internal Medicine 1, Ca’ Foncello Hospital, ULSS2 Marca Trevigiana, 31100 Treviso, Italy; francesco.cinetto@unipd.it; 11 Veneto Institute of Molecular Medicine (VIMM), 35129 Padua, Italy

**Keywords:** immune thrombocytopenia, splenectomy, prognostic factors

## Abstract

**Background/Objectives**: Splenectomy leads to a high rate of remission in chronic primary immune thrombocytopenia (ITP), but its unpredictable long-term positive outcomes and that it is a irreversible surgical approach discourage clinicians and patients. The identification of predictors of response may redefine the timing of splenectomy. In this retrospective, multicentric study we aimed to investigate clinical–histological predictors of splenectomy response in ITP patients and provide an easy-to-use score to predict splenectomy response in ITP. **Methods**: We considered a discovery set (*n* = 17) and a validation set (*n* = 30) of adult ITP patients, who underwent splenectomy for refractory disease in three Italian referral centers for ITP. **Results**: We found that the presence of autoimmune comorbidities, daily steroid dose prior to splenectomy, age at diagnosis and age at splenectomy were significantly associated with the outcome. Variables singly associated with an adverse outcome were combined into a clinical and a clinical–pathological score, allowing us to define a “high-risk” group which accounted for about 80% of the disease relapses observed in this cohort. At the same time, a certain clinical–pathological score indicated a “high-risk” group characterized by significantly poorer outcomes. Results were confirmed in the validation cohort. **Conclusions**: An integrated set of clinical and histological parameters may predict the response to splenectomy in ITP patients. While these findings provide valuable insights, they were derived from a small cohort of patients and therefore require validation in larger, more diverse populations to ensure their generalizability and robustness.

## 1. Introduction

Primary immune thrombocytopenia (ITP) is an acquired autoimmune disorder characterized by isolated thrombocytopenia (platelet count < 100 × 10⁹/L), driven by immune-mediated platelet destruction and impaired thrombopoiesis [1]. Its pathogenesis remains poorly understood, likely resulting from a complex interplay of humoral and cellular immune dysfunction, positioning ITP as a multifaceted clinical syndrome rather than a singular disease entity [1]. First-line treatments typically include corticosteroids and intravenous immunoglobulin (IVIg), achieving an initial response rate of 80–85%, though long-term responses decline to 20–30% [2,3]. For patients who relapse or are refractory, additional options include immunosuppressants such as rituximab and mycophenolate mofetil, thrombopoietin receptor agonists (TPO-RAs), Syk inhibitors like fostamatinib, and, in chronic cases, splenectomy [3,4,5,6]. Splenectomy offers the highest long-term response rates, with approximately 70% of patients maintaining remission over 20 years [7,8], underscoring the spleen’s central role in antiplatelet antibody production and platelet destruction [9]. Despite its efficacy, splenectomy is generally reserved for patients with refractory disease due to its associated risks, including infections and venous thromboembolism [9,10,11]. Furthermore, previous retrospective studies have failed to identify consistent preoperative or postoperative predictors of splenectomy response [9,12,13].

Building on insights from a 2021 pilot study [14], this study aimed to evaluate clinical, laboratory, and histological parameters in two cohorts of ITP patients (n_1_ = 17; n_2_ = 30) undergoing laparoscopic splenectomy (LS). Our goal was to identify potential predictors of splenectomy outcomes and propose a simple, clinically applicable scoring system to predict surgical response [15].

## 2. Materials and Methods

In our multicentric retrospective study, we first considered a cohort of 17 chronic ITP patients (discovery set) diagnosed and treated in a tertiary medical center and undergoing LS for refractory ITP over a 17-year period (February 2003–June 2020). The inclusion criteria were set as follows: (a) age at splenectomy of ≥16 years; (b) availability of clinical and laboratory data before/after splenectomy (e.g., age at diagnosis; presence of autoimmune comorbidities; pre-splenectomy medical therapies; platelet count on the day before and at the time of discharge after splenectomy); (c) availability of representative surgical spleen samples; (d) follow up ≥3 months after splenectomy. Surgery was performed, based on clinical judgment, in refractory cases, in patients with chronic ITP in need of chronic therapies. Based on ITP International Working Group (IWG) criteria [15], we identified two groups of patients: (i) responders, patients with persistent complete response (CR) or response (R) after surgery; (ii) non-responders, patients with loss of response (LR) or no response (NR) after surgery. The histological pattern of the removed spleens was evaluated in accordance with our previous findings [14].

Variables associated with surgical outcome (*p* < 0.05), even marginally (*p* < 0.1), were combined into a clinical score designed to assess the risk of ITP recurrence following splenectomy. The histological variables associated with non-responsiveness were also integrated into a clinical–histological score, aimed at assessing the risk of early recurrence following splenectomy. Each variable in the scores was arbitrarily assigned a value of 1. Data were validated in a second cohort of patients with the same inclusion criteria as the discovery set (*n* = 30), collected from 2 external Italian centers (*n* = 3; San Bortolo Hospital, Vicenza: *n* = 12; Policlinico Sant’Orsola–Malpighi, Bologna: *n* = 15) and 3 patients from the Internal Medicine Unit of the Padova University Hospital, Padova, over an 18-year period (October 2003–December 2021).

For histological and morphometric evaluation, all cases were analyzed at the Pathology Unit of Padova University Hospital (Padova–Italy) and representative samples were selected for further histological characterization. In particular, the following parameters were considered: (a) spleen weight; (b) presence of accessory spleens; (c) histological pattern; (d) white pulp (WP) lymphoid follicle (LF) density (i.e., number of LF per square unit area in mm^2^); (e) marginal zone (MZ) density (i.e., number of MZ per square unit area in mm^2^) and width (i.e., MZ/WP ratio); (f) T follicular helper (Tfh) cell density (i.e., number of PD1 Bcl6 double-positive cells within B cell follicles per high-power field [HPF]). Morphometric analysis and image acquisition were performed using the Leica DFC295 digital color camera and LAS X 1.4.6 software (Leica Microsystems, Milano–Italy).

Immunohistochemical analysis were performed as previously described [14]. Primary antibodies used for immunostains are detailed in the Supplementary Data.

For statistical analysis, in the discovery set, the distribution of quantitative variables was assessed by the Shapiro–Wilk test, and appropriate analysis for qualitative (Fisher’s exact test or Chi-squared test according to the frequency in the contingency tables) and quantitative (normally distributed variables: Student’s *t* test; non-normally distributed variables: Mann–Whitney U test) variables were applied. The variables significantly associated, or trending toward significance (*p* < 0.1), with non-responsiveness to splenectomy in univariate analyses were combined into a prognostic score. For this purpose, quantitative variables were transformed into dichotomous qualitative variables, with cutoff values determined through ROCs and Youden’s index. The composition of the predictive scores was designed to maximize the negative predictive value (NPV) associated with a ‘low-risk’ status. Relapse-free survival (RFS) was calculated using the Kaplan–Meier method and compared through the log-rank test. The statistical tests applied in the discovery set were used appropriately to evaluate the consistency of the results in the validation set.

Data analysis was performed using R (https://www.R-project.org, accessed on 29 February 2024) and Prism 10 (GraphPad.com), Boston, MA, USA). Differences between groups were considered statistically significant for *p*-values < 0.05.

## 3. Results

### 3.1. Clinical–Epidemiological Features of the Discovery Set

The discovery set included 12/17 (79.0%) females, with a median age at diagnosis of 32.4 years (standard deviation [SD]: 17.4 years) (Table 1). All patients were diagnosed with ITP and were refractory to first-line therapies. Pre-splenectomy therapies included steroids in all patients (median steroid dose: 37.5 mg daily); 5/17 (29.4%) received IVIg, 5/17 (29.4%) rituximab, and 6/17 (35.2%) TPO-RA. Overall, 16/17 (94.0%) patients were receiving chronic steroid therapy (median steroid dose: 25.0 mg daily) at the time of LS, and 6/17 (35.3%) patients were taking TPO-RA. In addition, a subset of patients had been treated with other drugs, alone or in combination (danazol or cyclophosphamide: one case each; vincristine: two cases; cyclosporin: two cases; mycophenolate mofetil: four cases; azathioprine: seven cases). Non-hematologic autoimmune comorbidities and/or high autoantibody titers were found in 6/17 (35.2%) cases (autoimmune hemolytic anemia-AHA, systemic lupus erythematous-SLE, Basedow’s disease, high-titer positivity for ANA autoantibodies, Sjögren syndrome, autoimmune polyendocrine syndrome type 2: one case each).

Splenectomy prompted a substantial clinical response in 15/17 (88.2%) cases, with CR in all 15 responding patients. Lack of response (NR) was reported in 2/17 (11.7%) cases, and LR after a transitory improvement in platelet count was reported in 5/15 (33.3%) cases (4 cases by the first 9 months, and 1 case after 115 months). The median post-splenectomy follow-up was 26 months (range: 3–158 months), with a 1- and 10-year RFS of 64.7% and 53.9%, respectively. One patient died at the follow-up (after 20 years from splenectomy) for unrelated causes. No significant complications related to splenectomy were reported.

### 3.2. Histopathological Features of Spleens from the Discovery Set

Spleens belonging to ITP patients in the discovery set had a median weight of 134.2 g (SD: 50.7 g), and accessory spleens were present in 6/17 (35.2%) cases. Microscopic examination (Table 2) identified three morphological patterns: (a) a hyperplastic white pulp (HWP) pattern in 9/17 (53%) cases, characterized by well-demarcated LFs and large germinal centers (GCs); (b) a non-activated white pulp (nAWP) pattern was in 7/17 (41.2%) cases, with many LFs without evidence of GCs; (c) a white pulp-depleted (WPD) pattern in 1/17 (5.9%) cases, characterized by marked lymphoid atrophy.

### 3.3. Clinical–Pathological Features and Response to Splenectomy

To investigate clinical–pathological features predictive of splenectomy outcome, we identified two subgroups in the discovery set: (i) responders, patients achieving CR or R after splenectomy, comprising 10/17 (58.9%) cases, and (ii) non-responders, displaying NR or LR after splenectomy (7/17 [41.0%] cases). Autoimmune comorbidities and/or high autoantibody titers were more frequently reported in non-responders vs. responders (5/7 [71.4%] non responders vs. 1/10 [10%] responders; *p* = 0.036). The steroid dosage before splenectomy was significantly higher in non-responders (median prednisone-equivalent dose: 40 mg daily among non-responders vs. 13.25 mg daily among responders; *p* = 0.011). In particular, a daily steroid dose ≥37.5 mg in the seven days prior to splenectomy was found to be statistically associated with non-response to splenectomy (4/7 [57.4%] non-responders vs. 0/10 responders; *p* = 0.014; likelihood ratio 2.8). The age at diagnosis of ITP but not age at splenectomy was significantly higher in non-responders (mean age at diagnosis: 51.5 years among non-responders vs. 27.7 years among responders, *p* = 0.04; mean age at splenectomy: 53 years among non-responders vs. 34 among responders, *p* = ns). Non-response to splenectomy was statistically associated with an age at diagnosis of ITP ≥ 40 years (5/7 [71.4%] non-responders vs. 2/10 [20%] responders; *p* = 0.033; likelihood ratio 3.5).

The ITP histological patterns did not show a correlation with the response to splenectomy, while some specific morphometric parameters were associated with the outcome. Tfh density was significantly higher among responders (mean Tfh density: 55 cells/HPF among responders vs. 26 cells/HPF among non-responders; *p* = 0.033) and a Tfh density < 36.5 cells/HPF was statistically associated with splenectomy failure (5/7 [71.4%] non-responders vs. 1/10 [10%] responders; *p* = 0.034, likelihood ratio 8.5). No differences in the MZ/WP ratio were observed between responders and non-responders. Moreover, MZ hypoplasia, defined as MZ/WP < 0.434, showed no significant association with the outcome, despite its higher prevalence among non-responders (5/7 (71.4%] vs. 4/10 [40%] responders; *p* = 0.33).

No other clinical, histological or morphometric variable correlated with the response to surgery (Table 3).

### 3.4. Multiparametric Analysis to Predict the Response to Splenectomy in the Discovery Set

Despite some clinical–pathological features being statistically associated with the response to splenectomy, none could independently predict the surgical outcome in a multivariate model. To enhance the predictive power of the model, the variables that demonstrated predictive power in univariate analysis were combined into two scores: (i) clinical (i.e., autoimmune comorbidities/high autoantibody titers; high pre-splenectomy steroid dosage [≥37.5 mg daily]; age at diagnosis ≥ 40 years), and (ii) clinical–pathological (i.e., fewer PD1-positive Tfh cells [Tfh density < 36.5 cells/HPF], and marginal zone atrophy [MZ/WP < 0.434], together with the aforementioned clinical variables). Each variable considered was arbitrarily assigned a value of 1.

**Figure 1:**
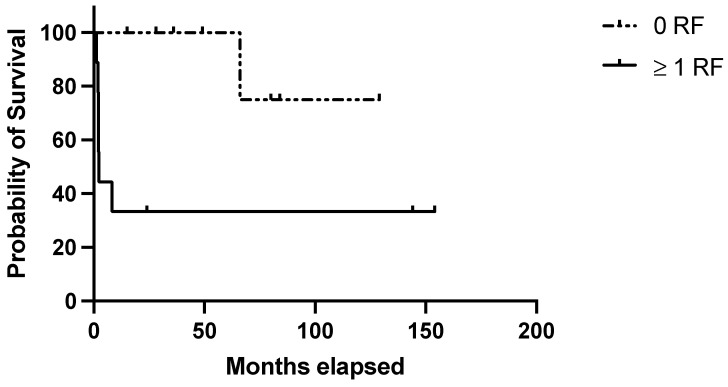
Evaluation of the clinical score in the discovery set. RFS in the discovery set based on the presence of no (=low risk) or 1–3 (=high risk) risk factors among the following: age at diagnosis ≥ 40 years; autoimmune comorbidities/high-titer autoantibodies; pre-splenectomy daily steroid dosage ≥ 37.5 mg.

**Figure 2:**
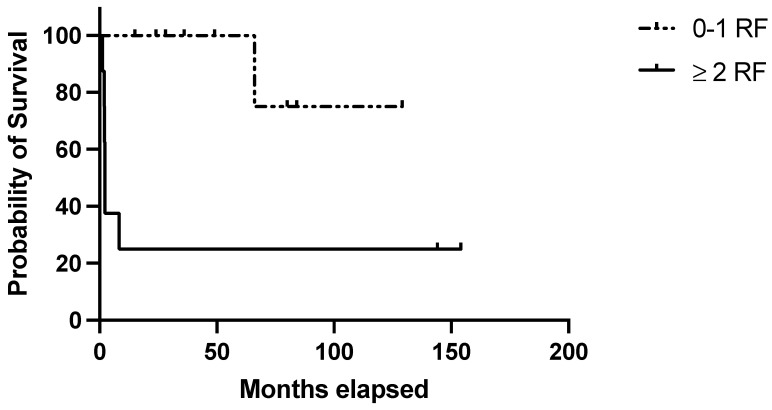
Evaluation of the clinical–pathological score in the discovery set. RFS in the discovery set based on the presence of 0–1 (=low risk) or 2–5 (=high risk) risk factors with at least 1 clinical risk factor (age at diagnosis ≥ 40 years; autoimmune comorbidities/high-titer autoantibodies; pre-splenectomy daily steroid dosage ≥37.5 mg) and at least 1 pathological negative predictive factor (Tfh density < 36.5 cells/HPF; MZ/WP < 0.434).

Regarding the clinical score, 9/17 (53%) patients in the discovery belonged to the “high-risk” group (i.e., ≥1 negative prognostic factor) and accounted for 77.7% of the disease relapses observed in this cohort (1- and 10-year RFS: 33.3% in high-risk patients vs. 100% and 75% in low-risk ones; *p* = 0.01) (Figure 1). Considering the clinical–pathological score, 8/17 (47.0%) belonged to the “high-risk” group (patients with at least one clinical risk factor and at least one pathological negative predictive factor) and were characterized by significantly poorer outcomes (1- and 10-year RFS: 25% in high-risk patients vs. 100 and 75% in low-risk ones; *p* = 0.003) (Figure 2). Table 4 summarizes the performance of the score in the discovery set.

### 3.5. Validation of the Results

The statistical analysis used in the discovery set was then applied to the validation set, consisting of 30 patients who underwent therapeutic splenectomy for chronic ITP across three centers. The sample size for the validation cohort was estimated based on an expected model sensitivity of 85%, an outcome prevalence of 40%, an acceptable margin of error (precision) of 5%, and a power of 80%. Using these parameters, the sample size calculation indicated that 30 patients would be sufficient to validate the model’s sensitivity.

Epidemiological, clinical, histological characteristics, and the follow-up period of the validation set were similar to those of the discovery set, with the exception of slightly different pre-splenectomy platelet counts (Table 5).

**Figure 3:**
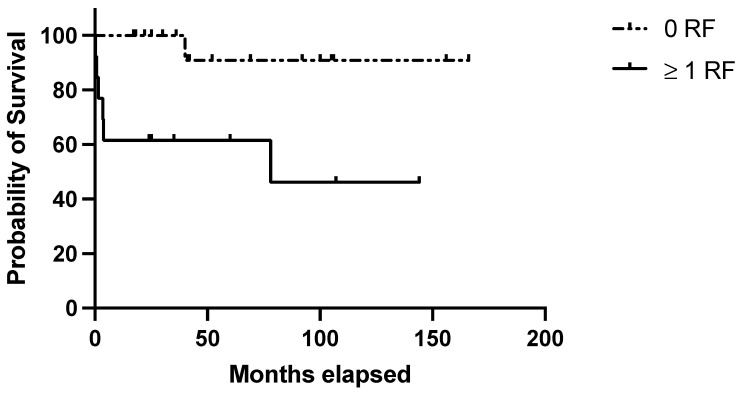
Evaluation of the clinical score in the validation set. RFS in the validation set based on the presence of no (=low risk) or 1–3 (=high risk) risk factors among the following: age at diagnosis ≥ 40 years; autoimmune comorbidities/high-titer autoantibodies; pre-splenectomy daily steroid dosage ≥ 37.5 mg.

The clinical score identified 12/30 (40%) cases at high risk of relapse, of which 6/12 (50%) actually relapsed. In the “low-risk” group (18/30 [60%] cases), only 1/18 (5.5%) experienced a relapse. Survival analysis confirmed lower RFS among patients featuring ≥ 1 adverse prognostic indicators (1 and 10-year RFS: 58,3% and 40.7% in the high risk group vs. 100% and 91% in the low risk one; *p* = 0.007) (Figure 3).

**Figure 4:**
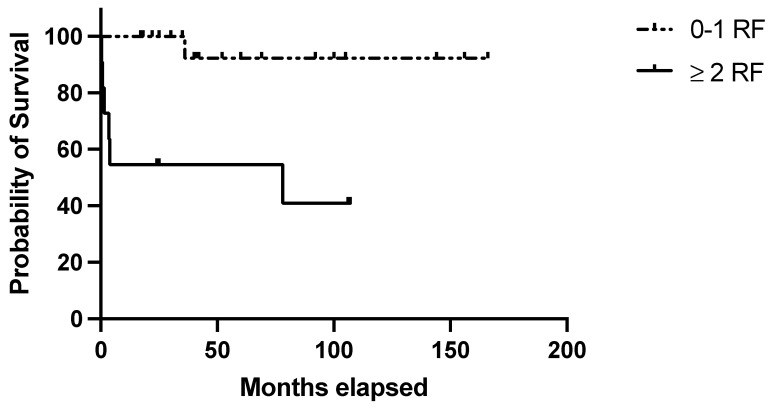
Evaluation of the clinical–pathological score in the validation set. RFS in the validation set based on the presence of 0–1 (=low risk) or 2–5 (=high risk) risk factors with at least 1 clinical risk factor (age at diagnosis ≥ 40 years; autoimmune comorbidities/high-titer autoantibodies; pre-splenectomy daily steroid dosage ≥ 37.5 mg) and at least 1 pathological negative predictive factor (Tfh density < 36.5 cells/HPF; MZ/WP < 0.434).

The clinical–histological score was applicable to all 30 cases and identified 11/30 (36.6%) cases at high risk of relapse, of which 6/11 (54.5%) relapsed (*p* = 0.004; Figure 4). Table 4 summarizes the performance of the score in the validation set.

## 4. Discussion

The variability in the natural history and therapeutic response of immune thrombocytopenic purpura (ITP) suggests that it represents a clinical syndrome with diverse pathogenic mechanisms rather than a singular disease entity [1,16]. The spleen plays a pivotal role in the pathogenesis of ITP, as demonstrated by the consistently high response rates to splenectomy reported in patients [7,8,9]. However, the use of splenectomy is limited by potential surgical complications, an increased risk of infections, and thrombotic events [9,10,11]. Therefore, identifying biological factors that predict response to splenectomy is essential for clinicians when discussing the risks and benefits of this procedure with patients. To address this need, we first identified clinical, laboratory, and histological parameters predictive of laparoscopic splenectomy outcomes in a discovery cohort of ITP patients and subsequently validated these parameters in an independent cohort. Consistent with previous studies, many commonly proposed predictors of splenectomy response—such as gender, disease duration, pre- and post-splenectomy platelet counts, severity of hemorrhagic symptoms, spleen size, and splenic follicular hyperplasia—demonstrated limited prognostic value on their own [12].

Conversely, we identified steroid dosage, the presence of autoimmune comorbidities, and age at diagnosis (≥40 years) as key factors that stratify ITP patients into distinct risk groups with varying post-splenectomy outcomes. Notably, the presence of one or more of these risk factors was associated with reduced 10-year relapse-free survival (RFS) following splenectomy, a finding that was validated in our independent cohort.

Among the parameters examined, age emerged as particularly significant. In our study, the median age at diagnosis for patients who achieved a complete response was 33 years, compared to 50 years for non-responders. This aligns with prior research suggesting that younger patients tend to have more favorable outcomes after splenectomy, though an exact age cutoff remains undefined [12,17].

One study highlighted that the likelihood of success and duration of response after splenectomy may decrease in patients over 65 years of age [17]. While we observed a relationship between age at splenectomy and surgical outcomes, we propose that age at diagnosis is a more reliable prognostic marker, as it is less influenced by non-surgical treatment availability and local healthcare policies.

Additionally, we found that a daily (prednisone-equivalent) steroid dose of ≥37.5 mg in the week preceding splenectomy was significantly associated with poorer surgical outcomes. While some studies have linked initial corticosteroid response to splenectomy efficacy, the evidence remains inconsistent. Notably, few investigations have specifically examined the impact of pre-surgical steroid dosage, which may more accurately reflect the disease’s intrinsic resistance to treatment and the need to sustain steroid therapy through multiple treatment lines as a bridge to splenectomy.

Although the response to medical treatment in primary and secondary ITP is generally similar, patients with concurrent autoimmune disorders tend to have poorer outcomes following splenectomy [18,19]. This is particularly evident in secondary forms of ITP involving defects in central tolerance, such as autoimmune lymphoproliferative syndrome, antiphospholipid syndrome (APS), and systemic lupus erythematosus (SLE). In cases like APS-associated ITP, rituximab is often preferred over splenectomy due to its superior response rates [20,21]. Therefore, the reduced response rates observed in our cohort with coexisting autoimmune conditions are not surprising and underscore the complexity of selecting appropriate candidates for splenectomy.

Histological evaluations of the spleens confirmed the presence of various patterns; however, none showed a clear correlation with splenectomy outcomes. Interestingly, marginal zone hypoplasia and reduced T follicular helper (Tfh) cell density emerged as potential predictors, possibly indicating cases where the spleen is not the primary site of the pathophysiological process. Marginal zone hypoplasia may reflect a deficiency in key B cell subsets responsible for capturing and presenting antigens to follicular dendritic cells, thereby affecting antibody responses. Similarly, a reduction in Tfh cells could impair B cell activation, expansion, differentiation, and germinal center formation within the spleen [22,23].

Regarding the observed differences in pre-splenectomy platelet counts between the discovery and validation cohorts, we carefully ruled out significant biases in our analysis. The decision to proceed with splenectomy is largely influenced by the clinical course, previous therapeutic responses, and patient expectations, rather than baseline platelet counts alone.

Our patient selection strategy for splenectomy in ITP effectively identifies those at low risk of relapse, as demonstrated by its high negative predictive value, making it a valuable tool for real-world clinical decision-making. Incorporating a pathological score into clinical risk factors enhanced screening accuracy. Notably, these post-splenectomy findings could be translated into pre-splenectomy evaluations, as evidence suggests that variations in circulating Tfh cells may correlate with the clinical severity of autoimmune diseases [24,25]. In the context of ITP, this may provide insights into the likelihood of responding to splenectomy.

We observed a lower long-term response to splenectomy in the discovery cohort compared to the validation cohort and the existing literature. Previous studies have suggested that prior lines of therapy may negatively impact splenectomy outcomes [26]. Although we did not detect significant differences between our cohorts, we noted a higher use of immunosuppressants such as azathioprine and ciclosporine in the discovery set before splenectomy. This observation warrants further evaluation in larger patient cohorts to better understand its potential impact on surgical outcomes.

The primary limitation of our study is the relatively small size of the cohorts analyzed and the lack of multivariate analyses. We acknowledge that broader external validation of the proposed clinical score across diverse populations is necessary to confirm its reliability and applicability. Additionally, it will be important to investigate whether other factors, such as the use of thrombopoietin receptor agonists (TPO-RAs) as a bridge to surgery or the requirement for multiple intravenous immunoglobulin (IVIg) administrations, could further refine the identification of patients less likely to benefit from splenectomy.

In conclusion, we propose a simple, easy-to-use clinical score to improve the selection of ITP patients most likely to benefit from splenectomy. While this approach offers a valuable tool for personalized treatment in ITP patients, its validation in larger, more representative patient cohorts is necessary to confirm its broader applicability and reliability.

## Figures and Tables

**Table 1:** Clinical–epidemiological features of the discovery set (*n* = 17).

Variable	Discovery Set (*n* = 17)
Gender *n* (%)	
Female	12 (79.0)
Male	5 (21.0)
Median age at diagnosis of ITP (years)	32.4 ± 17.4
Median platelet count (×10^9^/L)	
ITP diagnosis	20 (IQR: 10.5–35.5)
Pre-splenectomy ^1^	32 (IQR:22.5–46.0)
Post-splenectomy ^2^	323 (IQR: 179.0–428.5)
Thrombocytopenia-related symptoms *n* (%)	
Minor ^3^	13 (52.0)
Major ^4^	3 (12.0)
Autoimmune comorbidities *n* (%)	6 (35.2)
Steroids before splenectomy *n* (%)	16 (94)
Median steroid dose pre-splenectomy (mg/die)	25 (IQR: 10.0–44.0) (range: 0–75)
TPO-RA before splenectomy *n*, (%)	6 (35.2)
Median age at splenectomy (years)	36 ± 15.6 (range: 22–68)
Splenectomy outcome ^5^ *n* (%)	
CR	15/17 (88.2)
R	0 (0.0)
NR	2/17 (11.7)
Loss of Response—LR *n* (%)	5/15 (33.3)
Median time to LR, months (range)	2.5 (2–115)

^1^ Lowest platelet count in the month before splenectomy. ^2^ Second highest platelet count in the two months following splenectomy. ^3^ Muco-cutaneous purpura and/or epistaxis. ^4^ Critical site bleeding (intracranial, intraspinal, intraocular, intra-articular, intramuscular, pericardial, or retroperitoneal) and/or bleeding that resulted in a ≥20 g/L Hb reduction or need for transfusion of at least 2 red blood cell units. ^5^ IWG criteria.

**Table 2:** Histopathological features of spleens of the discovery set (*n* = 17).

Variable	Discovery Set (*n* = 17)
Spleen weight (g)	134.2 ± 50.7
Accessory spleens *n* (%)	6/17 (35.3%%)
Histological pattern, *n* (%)	
HWP	9/17 (47.1%)
nAWP	7/17 (41.2%)
WPD	1/17 (5.9%)
LF density (no/mm^2^)	0.6 ± 0.3
WP diameter (μm)	372.5 ± 75.1
MZ thickness (μm)	151.9 ± 77.0
MZ/WP ^1^	0.42 (IQR: 0.32–0.50)
Tfh cell density (no/HPF)	40.0 (IQR: 24.0–56.0)

Abbreviations: HWP, hyperplastic white pulp; nAWP, non-activated white pulp; WPD, white pulp depleted; LF, lymphoid follicles; WP, white pulp; MZ, marginal zone; Tfh, T follicular helper (PD1+ cells). ^1^ Surrogate parameter to evaluate MZ expansion.

**Table 3:** Clinical–pathological predictors in the discovery set (*n* = 17).

Variable	Responders (*n* = 10)	Non-Responders (*n* = 7)	(*p*-Value)
Gender, *n* (%)			
Female	7 (70)	5 (71.4)	n.s.
Male	3 (30)	2 (28.6)	
Mean age at diagnosis (years)	27.5 ± 11.7	51 ± 20.3	0.046
Mean platelets count (×10^9^/L)			
ITP diagnosis	25 (IQR: 10.5–32.5)	19 (IQR: 10.3–64.5)	n.s.
Pre-splenectomy ^1^	29 (IQR: 21.5–63)	31.5 (IQR: 23.3–41.5)	n.s.
Autoimmune comorbidities. *n* (%)			
Absent	9 (88.2)	2 (28.6)	0.036
Present	1 (11.8)	5 (71.4)	
Median steroid dose			
Pre-splenectomy, mg/die	13.5 (IQR: 5.6–25)	40 (IQR: 15.6–50.0)	0.011
Median duration of illness pre-splenectomy (months)	30 (IQR: 10–79)	23 (IQR: 6–41)	n.s.
Mean age at splenectomy (years)	27 ± 13.9	51 ± 10.9	0.046
Histological pattern, *n* (%)			
HWP	6 (60)	3 (30)	
nAWP	4 (40)	3 (30)	n.s.
WPD	0 (0)	1 (10)	
LF mean density (no/mm^2^)	0.6 ± 0.3	0.5 ± 0.3	n.s.
WP mean diameter (μm)	388.9 ± 70.8	339.8 ± 82.2	n.s.
MZ hypoplasia, *n* (%) ^2^			
Present	4 (40)	5 (71.4)	0.33
Absent	6 (60)	2 (28.5)	
Tfh expansion ^3^ *n*, (%)			
Present	9 (90)	2 (28.5)	0.034
Absent	1 (10)	5 (71.4)	

^1^ Minimum platelet count in the month prior to splenectomy. ^2^ Defined as an MZ/WP ratio < 0.434. ^3^ Defined as a Tfh density ≥ 36.5 cells/high-power field.

**Table 4:** Sensitivity, specificity, PPV, NPV and accuracy of the score in the discovery and validation sets.

	Sensitivity	Specificity	PPV	NPV	Odd Ratios	C Index	*p*
Discovery set Clinical factors	85.7	70	66.6	87.5	14	76.5	0.049
Discovery set Clinical–pathological factors	85.7	80	74.8	88.9	24	82.4	0.015
Validation set Clinical factors	85.7	74	50	94.4	17	76.7	0.008
Validation set Clinical–pathological factors	85.7	78.3	54.5	94.7	21.6	80	0.004

**Table 5:** Clinical–pathological features of the discovery (*n* = 17) and the validation set (*n* = 30).

Variable	Discovery Set (*n* = 17)	Validation Set (*n* = 30)	(*p*-Value)
Gender, *n* (%)			
Female	12 (79.0)	21 (70)	n.s.
Male	5 (29.4)	9 (30)	
Mean age at diagnosis of ITP (yrs)	32.4 ± 17.4	32 ± 16.6	n.s.
Mean platelets count (×10^9^/L)			
ITP diagnosis	20 (IQR: 10.5–35.5)	32 (IQR: 9.5–51.5)	n.s.
Pre-splenectomy ^1^	32 (IQR: 22.5–46.0)	9 (IQR: 4.0–12.0)	*p* < 0.001
Post-splenectomy ^2^	323 (IQR:179.0–428.5)	371 (IQR: 162.0–535.0)	n.s.
Autoimmune comorbidities (%)	6 (35.0)	4 (13.3)	n.s.
TPO-RA before splenectomy (%)	6 (35.3%)	6 (20)	n.s.
Median duration of illness before splenectomy (months)	28 (IQR: 10–49)	32 (IQR:15–87)	n.s.
Treatment lines, median (range)	3.5 (1–9)	2.5 (1–9)	n.s.
Mean age at splenectomy, yrs (range)	35.7 ± 16.2 (19–68)	38 ± 18.9 (18–77)	n.s.
Response to Splenectomy (%) ^3^			n.s.
CR	15/17 (88.2)	29 (94.8)	
R	0 (0.0)	0 (0)	
NR	2/17 (11.7)	1 (3.3)	
Loss of Response—LR, *n* (%)	5/15 (33.3)	6/30 (20)	n.s.
Splenectomy outcome, *n* (%)			
Responders	15 (88.2)	23 (76.6)	n.s.
Non-responders	2 (11.7)	7 (23.3)	
Post-splenectomy RFS, *n* (%)			
1-year	64.7 (C.I 17.6–26.9)	73.8 (C.I. 9.3–18.8)	n.s.
10-years	53.9 (C.I. 22–29)	70.3 (C.I. 15.3–25.5)	n.s.
Median post-splenectomy follow-up	32, IQR: 2–106 (3–164)	28, IQR: 6–8 (3–171)	n.s
months (range)			
Histological pattern, *n* (%)			
HWP	9/17 (47.1%)	19/30 (63)	n.s.
nAWP	7/17 (41.2%)	8/30 (26.6)	
WPD	1/17 (5.9%)	3/30 (10)	
Median MZ/WP	0.42 (IQR: 0.32–0.50)	0.47 (IQR: 0.35–0.49)	n.s.
Median Tfh density (no/HPF)	40.0 (IQR: 24.0–56.0)	31.5 (IQR:10.7–52.3)	n.s.

^1^ Minimum platelet count in the month prior to splenectomy. ^2^ Second highest platelet count in the two months after splenectomy. ^3^ IWG criteria.

## Data Availability

Data underlying this article cannot be shared publicly because of the privacy of individuals who participated in the study. The data can be shared by the corresponding authors upon reasonable request.

## Supplementary Materials

The following supporting information can be downloaded at: https://www.mdpi.com/article/10.3390/jcm14010030/s1, Primary antibodies used for immunostains.
