# Supplementary material for: Predictors of Splenectomy Response in Immune Thrombocytopenia: A Multicentric Italian Study
Source: J Clin Med. 2024 Dec 25;14(1):30. doi: 10.3390/jcm14010030 (PMC11722461; doi:10.3390/jcm14010030)
Supplement: Supplementary file 1 [file jcm-14-00030-s001.zip › jcm-3330300-supplementary.pdf]

Primary antibodies used for immunostains

anti-CD4 (clone 4B12; Dako); anti-CD8 (clone C8/144B; Dako); anti-GATA3 (clone L50-823; Cell Marque); anti-t-Bet (clone 4B10; Abcam); anti-Foxp3 (clone 236A/E7; Leica Biosystems); anti-CD138 (clone MI15; Dako); anti-CD10 (clone 56C6; Dako); anti-CD68 (clone PG-M1; Dako); anti PD1 (clone NAT105; Abcam); anti-NMDA (clone 253A; Abcam).
